# Microwave-Assisted Cu-Catalyzed Diaryletherification for Facile Synthesis of Bioactive Prenylated Diresorcinols

**DOI:** 10.3390/molecules28010062

**Published:** 2022-12-21

**Authors:** Seoyoung Jo, Bohun Kang, Jong-Wha Jung

**Affiliations:** 1 College of Pharmacy, Research Institute of Pharmaceutical Sciences, Kyungpook National University, Daegu 41566, Republic of Korea; 2 Vessel-Organ Interaction Research Center, Kyungpook National University, Daegu 41566, Republic of Korea

**Keywords:** Cu-catalyzed diaryletherification, microwave-assisted cross-coupling, prenylated diresorcinols, diorcinol I, leotiomycene B

## Abstract

Prenylated diresorcinols exhibit various bioactivities, including cytotoxic, antibacterial, and antifungal activities. Therefore, establishing facile and efficient synthetic routes for prenylated diresorcinols facilitates their development as chemical probes or drugs with a novel mode of action. In this study, microwave-assisted copper catalysis was explored as a cost-effective and environmentally friendly method for the cross-coupling of sterically hindered *ortho*-prenylated phenols and aryl halides to produce bioactive prenylated diresorcinols, diorcinol I and leotiomycene B. Notable advantages of microwave-assisted catalysis include not only operational simplicity and rapid heating but also shorter reaction times and higher chemical yields. In addition, highly regioselective prenylation of phenol was achieved for the preparation of *ortho*-prenyl phenol via directed lithiation and subsequent alkylation. This study provides valuable insights for the preparation of other bioactive prenylated diresorcinols. Furthermore, considering that prenylated benzenoids are biosynthetic precursors of various polycyclic natural products, this synthetic route could be expanded to more complex bioactive compounds possessing diaryl ethers.

## 1. Introduction

**Figure 1:**
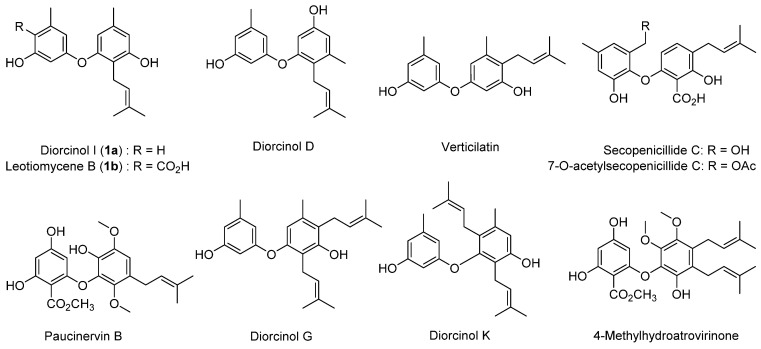
Bioactive prenylated diresorcinols.

Prenylated phenols are ubiquitous in nature. Their framework comprises a single or multiple benzene scaffolds, and their unique structure and various biological activities have attracted considerable attention [1,2]. Among the bioactive prenylated phenols, our interests lie in prenylated diresorcinols. Figure 1 shows the chemical structures of selected prenylated diresorcinols that exhibit various bioactivities. Diorcinol I (**1a**) and leotiomycene B (**1b**) are structurally related natural products. Diorcinol I (**1a**) exhibits cytotoxic [3] and antibacterial effects [4], while leotiomycene B (**1b**) inhibits bacterial quorum sensing [5]. Diorcinol D and verticilatin are fungal metabolites that are structurally related to diorcinol I (**1a**), differing only in the position of the prenyl group. Diorcinol D displays cytotoxic [6,7,8], antibacterial [4,9], and antifungal activities [10,11]. Verticilatin also exhibits various bioactivities related to Alzheimer’s disease, cancer [12] and bacterial infection [4]. Secopenicillide C and 7-O-acetylsecopenicillide C are antibacterial metabolites isolated from fungi [13]. Paucinervin B is an apoptotic compound from plants [14,15]. Diorcinol G and K are bisprenylated diresorcinols isolated from fungi, which show cytotoxic [3] and antibacterial [16] activities, respectively. 4-Methylhydroatrovirinone [17] is a reduced form of atrovirinone that exhibits antimicrobial, cytotoxic, and anti-inflammatory activities [18,19].

The prenyl group plays an important role in the bioactivity of prenylated diresorcinols. For example, recent structural inspection and hierarchical clustering suggested that the prenyl group as a side chain substituent of diresorcinols is essential for the antibacterial activity [20].

Establishing facile and efficient synthetic routes to bioactive prenylated diresorcinols facilitates their development as chemical probes or drugs with a novel mode of action. For the construction of the diresorcinol framework, diaryl ether synthesis is a pivotal step. Diaryl ethers have been of long-standing interest to synthetic organic chemists. In recent decades, metal-catalyzed cross-coupling reactions have been extensively utilized for the synthesis of diaryl ethers in academic, as well as industrial, settings. Palladium catalysis is arguably the leading method employed for efficient and versatile cross-couplings, including diaryletherification [21]. Copper catalysis is another effective method for cross-coupling reactions with a long history [22,23]. Conventional copper-catalyzed cross-coupling, namely the Ullmann reaction, suffers several disadvantages including the stoichiometric use of copper catalysts, harsh conditions, and a limited reaction scope. A breakthrough in copper catalysis for cross-coupling reactions in recent decades entails the development of catalytic systems using bidentate ligands, which allows for the catalytic use of copper catalysts, mild conditions, and a broad reaction scope including sterically hindered substrates. Furthermore, green methodologies for copper catalysis, including appropriate metal/ligand systems, the use of water and ionic liquids, solvent-free conditions, and microwave/ultrasound-assisted protocols, have been developed [24].

Herein, we report a microwave-assisted copper-catalyzed cross-coupling of a sterically hindered *ortho*-prenylated phenol and aryl halides. This catalytic system was applied to the synthesis of two bioactive prenylated diresorcinols, diorcinol I (**1a**), and leotiomycene B (**2a**).

## 2. Results and Discussion

**Figure 2:**
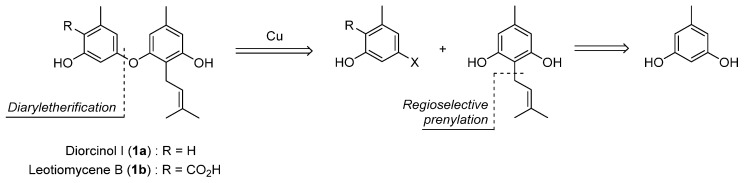
Retrosynthetic analysis of diorcinol I and leotiomycin B.

Our research focusing on bioactive prenylated diresorcinols necessitated the preparation of diresorcinols via diaryletherification. During screening for an appropriate catalytic system, we became interested in copper catalysis as it was a cost-effective and environmentally friendly method for cross-coupling. As shown in Figure 2, the prenyl group of diorcinol I (**1a**) and leotiomycene B (**1b**) are located immediately next to the diaryl ether bond, and it was envisaged that both compounds could be synthesized in parallel via the cross-coupling of the corresponding aryl halides and prenylated diresorcinol. Using aryl halides as a coupling partner of the prenylated phenol is advantageous due to their commercial availability and cost-efficiency. Preparation of the requisite prenylated resorcinol was envisaged to occur via regioselective *C*-alkylation of resorcinol.

We initially attempted the direct prenylation of commercially available phenol **2** under various conventional conditions, including the use of K_2_CO_3_ and NaH in aprotic nonpolar solvents [25]. However, these reactions provided a mixture of regioisomers, and isolation of the desired *ortho*-prenylated phenol using conventional chromatography was challenging and inefficient. Thus, we explored an alternative synthetic route via *ortho*-metalation employing a directing group [26,27].

**Scheme 1:**
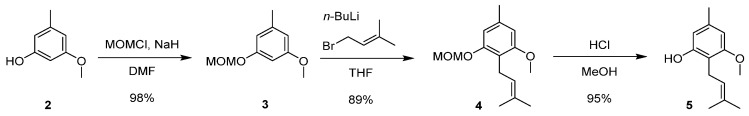
Preparation of prenylated phenol **5**.

As shown in Scheme 1, the synthesis commenced with the preparation of prenylated phenol **5**. Commercially available phenol **2** was protected with a methoxymethyl (MOM) ether group, and the resultant MOM ether served as a directing group. MOM-protected **3** was lithiated using *n*-BuLi in situ, and then prenylated with 1-bromo-3-methyl-2-butene to provide compound **4** in a highly regiospecific manner. Notably, deprotonation of the benzylic proton did not compete with directed lithiation. The MOM group was then cleaved under acidic conditions to afford the desired prenylated phenol **5**.

Next, we optimized the cross-coupling reaction between prenylated phenol **5** and aryl halide **6a** (Table 1). The initially attempted copper-catalyzed diaryletherification condition under microwave irradiation [28] did not provide the desired product **7a** (Table 1, entry 1). Thus, we explored copper catalytic systems comprising various bidentate ligands [22]. As the cross-coupling reaction may be impeded by the steric hindrance imposed by the *ortho*-prenyl group, we attempted a catalytic system comprising CuI, picolinic acid, and K_3_PO_4_, previously applied for the synthesis of sterically hindered diaryl ethers [29]. Although this catalytic system has been successfully applied for the cross-coupling of various sterically hindered *ortho*-methyl or methoxy phenols, it failed to provide **7a** under the reported thermal conditions (entry 2). Inspired by earlier studies on microwave-assisted copper catalysis for diaryletherification [28,30,31,32], we explored the application of microwaves in copper and bidentate ligand systems to promote the cross-coupling reaction. Gratifyingly, **7a** was formed under microwave irradiation in a relatively shorter reaction time (entries 3–5) compared to the condition with thermal heating (0.5 h vs. 24 h). Notably, the amount of unidentified side products increased when a higher temperature (entry 4 vs. entry 5) or prolonged reaction time (data not shown) were employed. Pyrrole-2-carboxylic acid, an alternative ligand to the structurally similar picolinic acid [29], proved to be ineffective in this case (entry 6). Although **7a** was obtained by the condition above, the low to moderate yields prompted us to explore other catalytic systems using second-generation bidentate ligands [22]. Several ligands were screened to arrive at following the optimized conditions: CuI, picolinamide ligand [33], and K_3_PO_4_ in acetonitrile at 120 °C under microwave irradiation for 0.5 h (entry 10). Notably, the optimized conditions using microwave irradiation were found to be more efficient than the reported conditions employing thermal heating [33] in terms of chemical yield and reaction rate (entry 7 vs. entries 8–10).

**Scheme 2:**
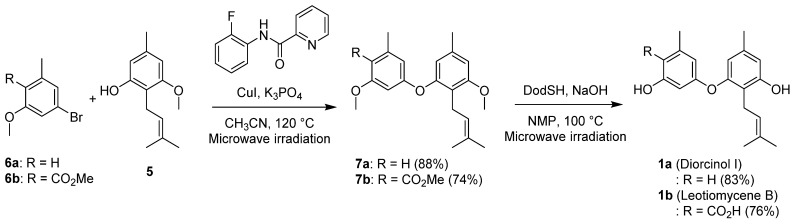
Synthesis of diorcinol I and leotiomycene B.

With the requisite diaryl ether **7a** in hand, the *O*-demethylation reaction was conducted according to a reported procedure [34] with slight modification. As shown in Scheme 2, compound **7a** was treated with an odorless long-chain thiol under microwave irradiation and basic conditions to cleave the two methyl groups in **7a**, affording diorcinol I (**1a**) in high yield. Notably, microwave irradiation improved the chemical yield of the *O*-demethylation reaction. Similarly, leotiomycene B was synthesized using the same protocol, except that aryl bromide **6b** was used instead of **6a** (Scheme 2). Compound **6b** was cross-coupled with prenylated phenol **5** under the optimized condition to afford diaryl ether **7b**. Notably, the reactions under microwave irradiation during the synthesis were conducted with 10 mL vessels limiting the total volume of reaction mixtures up to 7 mL due to the instrumental setting. Finally, global *O*-demethylation of the three methyl groups in **7b** provided leotiomycene B (**1b**). To the best of our knowledge, this is the first total synthesis of leotiomycene B. The NMR data of synthetic **1a** and **1b** are in good agreement with the reported data for diorcinol I [3] and leotiomycene B [5] (Tables S1 and S2, Supplementary Materials).

## 3. Materials and Methods

### 3.1. General Experimental Details

All reagents were purchased from commercial suppliers and used without further purification, unless stated otherwise. The solvents used for the chemical reactions were dried over appropriate drying agents or distilled prior to use. Air and moisture-sensitive experiments were conducted under an argon atmosphere in oven-dried glassware with magnetic stirring unless stated otherwise. Microwave irradiation reactions were performed using a CEM Discover SP instrument (Matthews, NC, USA). Analytical thin-layer chromatography was performed on Merck silica gel 60 F254 plates (Darmstadt, Germany), and flash column chromatography was performed using Biotage Isolera (Uppsala, Sweden). ^1^H and ^13^C NMR spectra were acquired on a Bruker Avance-500 spectrometer (Billerica, MA, USA) in deuterated solvent using Me_4_Si as the internal standard. Mass spectra were obtained using an Advion Expression CMS (Ithaca, NY, USA), and high-resolution mass spectra were recorded on a JEOL JMS-700 mass spectrometer (Tokyo, Japan) at the Daegu branch of the Korean Basic Science Institute.

### 3.2. Preparation of **5**

3-Methoxy-5-methylphenol (**2**, 1.0 g, 7.24 mmol) was dissolved in DMF (7 mL), and NaH (60% in mineral oil, 868 mg, 21.7 mmol) was added to the solution at 0 °C. After stirring for 10 min, chloromethyl methyl ether (0.825 mL, 10.86 mmol) was added to the resulting mixture. The reaction mixture was stirred at room temperature for 2 h and then quenched with water. The mixture was extracted using ethyl acetate and the combined organic phase was dried over anhydrous MgSO_4_ and filtered. Volatiles were removed under reduced pressure, and the residue was purified by SiO_2_ chromatography to afford **3** (1.3 g, 98%) as a yellow oil; Rf 0.45 (*n*-hexane:ethyl acetate = 9:1); ^1^H NMR (500 MHz, Chloroform-*d*) δ = 6.48 (s, 1H), 6.43 (s, 1H), 6.40 (s, 2H), 5.15 (s, 2H), 3.78 (s, 3H), 3.48 (s, 3H), 2.31 (s, 3H) ppm; ^13^C NMR (126 MHz, Chloroform-*d*) δ = 160.71, 158.38, 140.39, 109.28, 108.52, 99.76, 94.51, 56.10, 55.34, 21.89 ppm; LR-ESI-MS *m/z* 183 [M + H]^+^.

To a stirred solution of **3** (300 mg, 1.65 mmol) in THF (6 mL), *n*-BuLi (2.5 M in hexane, 0.8 mL, 2.0 mmol) was added at 0 °C. After stirring for 1 h at 0 °C, 1-bromo-3-methyl-2-butene (0.192 mL, 1.65 mmol) was added to the mixture at 0 °C. The reaction mixture was stirred for 2 h at room temperature and then quenched with aqueous NH_4_Cl. The mixture was extracted using ethyl acetate and the combined organic phase was dried over anhydrous MgSO_4_ and filtered. Volatiles were removed under reduced pressure, and the residue was purified by SiO_2_ chromatography to afford **4** (368 mg, 89%) as a colorless oil; Rf 0.40 (*n*-hexane:ethyl acetate = 15:1); ^1^H NMR (500 MHz, Chloroform-*d*) δ = 6.55 (s, 1H), 6.40 (s, 1H), 5.20 (t, *J* = 7.2 Hz, 1H), 5.17 (s, 2H), 3.80 (s, 3H), 3.47 (s, 3H), 3.33 (d, *J* = 7.1 Hz, 2H), 2.31 (s, 3H), 1.77 (s, 3H), 1.65 (s, 3H) ppm; ^13^C NMR (126 MHz, Chloroform-*d*) δ = 158.04, 155.51, 136.90, 130.92, 123.31, 116.40, 107.93, 105.81, 94.54, 56.09, 55.83, 25.96, 22.38, 22.04, 17.86 ppm; LR-ESI-MS *m/z* 249 [M − H]^−^_._

To a stirred solution of **4** (368 mg, 1.47 mmol) in MeOH (10 mL), c-HCl (0.48 mL) was added at 0 °C for 1 h. After stirring for 2 h, the reaction was quenched with aqueous NaHCO_3_. The mixture was extracted using ethyl acetate and the combined organic phase was dried over anhydrous MgSO_4_ and filtered. Volatiles were removed under reduced pressure, and the residue was purified with SiO_2_ chromatography to afford **5** (287 mg, 95%) as a colorless oil; Rf 0.26 (*n*-hexane:ethyl acetate = 15:1); ^1^H NMR (500 MHz, Chloroform-d) δ = 6.31 (s, 2H), 5.22 (t, *J* = 7.1 Hz, 1H), 5.17 (s, 1H), 3.80 (s, 3H), 3.37 (d, *J* = 7.2 Hz, 2H), 2.28 (s, 3H), 1.80 (s, 3H), 1.73 (s, 3H) ppm; ^13^C NMR (126 MHz, Chloroform-d) δ = 157.87, 155.25, 137.43, 134.17, 122.44, 112.24, 109.58, 104.38, 55.87, 25.94, 22.17, 21.69, 17.95 ppm; LR-APCI-MS *m/z* 207 [M + H]^+^_._

### 3.3. Synthesis of **7a** and **7b**

Prenylated phenol **5** (190 mg, 0.92 mmol) and 1-bromo-3-methoxy-5-methylbenzene (**6a**, 0.134 mL, 0.92 mmol) were dissolved in acetonitrile (4 mL). CuI (18 mg, 0.092 mmol), *N*-(2-fluorophenyl)picolinamide (20 mg, 0.092 mmol), and K_3_PO_4_ (390 mg, 1.86 mmol) were added to the solution. The reaction mixture was stirred for 30 min at 120 °C under microwave irradiation and then quenched with water after cooling. The mixture was extracted using ethyl acetate and the combined organic phase was dried over MgSO_4_ and filtered. Volatiles were removed under reduced pressure, and the residue was purified by SiO_2_ chromatography to afford **7a** (264 mg, 88%) as a yellow oil; Rf 0.45 (*n*-hexane:ethyl acetate = 15:1); ^1^H NMR (500 MHz, CDCl_3_) δ = 6.49 (s, 1H), 6.41 (s, 1H), 6.37 (s, 1H), 6.30 (s, 2H), 5.18 (m, 1H), 3.84 (s, 3H), 3.74 (s, 3H), 3.28 (d, J = 7.2 Hz, 2H), 2.26 (s, 3H), 2.26 (s, 3H) 1.67–1.56 (m, 6H) ppm; ^13^C NMR (125 MHz, CDCl_3_) δ = 160.70, 159.37, 158.47, 154.60, 140.37, 137.13, 131.37, 122.73, 119.42, 113.33, 110.85, 108.87, 107.49, 101.03, 55.84, 55.43, 25.92, 22.74, 21.81, 21.72, 17.80 ppm; LR-APCI-MS *m/z* 327 [M + H]^+^_._

Prenylated phenol **5** (311 mg, 1.51 mmol) and methyl 2-methoxy-4,6-dimethylbenzoate (**6b**, 391 mg, 1.51 mmol) were dissolved in acetonitrile (4 mL). CuI (29 mg, 0.151 mmol), *N*-(2-fluorophenyl)picolinamide (33 mg, 0.151 mmol), and K_3_PO_4_ (633 mg, 3.02 mmol) were added to the solution. The reaction mixture was stirred for 30 min at 120 °C under microwave irradiation and then quenched with water after cooling. The mixture was extracted using ethyl acetate and the combined organic phase was dried over MgSO_4_ and filtered. Volatiles were removed under reduced pressure, and the residue was purified by SiO_2_ chromatography to afford **7b** (430 mg, 74%) as a yellow oil; Rf 0.17 (*n*-hexane:ethyl acetate = 15:1); ^1^H NMR (500 MHz, Chloroform-d) δ = 6.52 (s, 1H), 6.37 (s, 1H), 6.35 (s, 1H), 6.25 (s, 2H), 5.15 (t, *J* = 7.3 Hz, 1H), 3.89 (s, 3H), 3.85 (s, 3H), 3.74 (s, 3H), 3.26 (d, *J* = 7.2 Hz, 2H), 2.27 (s, 3H), 2.22 (s, 3H), 1.61 (s, 3H), 1.60 (s, 3H) ppm; ^13^C NMR (126 MHz, Chloroform-d) δ 168.84, 160.16, 158.51, 158.19, 154.11, 138.27, 137.34, 131.57, 122.55, 119.54, 117.72, 113.40, 110.95, 107.85, 99.06, 56.12, 55.85, 52.28, 25.92, 22.73, 21.71, 19.80, 17.80 ppm; LR-ESI-MS *m/z* 385 [M + H]^+^. 

### 3.4. Synthesis of Diorcinol I (**1a**) and Leotiomycene (**1b**)

To a stirred solution of **7a** (182 mg, 0.55 mmol) in N-methyl-2-pyrrolidone (NMP) (3 mL), 1-dodecanethiol (0.39 mL, 1.65 mmol) and NaOH (132 mg, 3.3 mmol) were added. The reaction mixture was stirred for 1.5 h at 100 °C under microwave irradiation and then quenched with 1 N HCl (aq.) after cooling. The mixture was extracted using ethyl acetate and the combined organic phase was dried over MgSO_4_ and filtered. Volatiles were removed under reduced pressure, and the residue was purified by SiO_2_ chromatography to afford diorcinol I (**1a**, 132 mg, 83%) as a yellow oil; Rf 0.35 (*n*-hexane:ethyl acetate = 3:1); ^1^H NMR (500 MHz, DMSO-d6) δ = 9.45 (s, 1H), 9.35 (s, 1H), 6.46 (s, 1H), 6.27 (s, 1H), 6.17 (s, 1H), 6.14 (s, 1H), 6.03 (s, 1H), 5.13 (t, *J* = 7.4 Hz, 1H), 3.11 (d, *J* = 7.3 Hz, 2H), 2.17 (s, 3H), 2.15 (s, 3H), 1.58 (s, 3H), 1.58 (s, 3H) ppm; ^13^C NMR (126 MHz, DMSO-d6) δ = 159.35, 158.76, 156.65, 154.62, 140.21, 136.77, 130.48, 123.24, 117.38, 112.13, 111.95, 110.56, 108.89, 101.71, 25.98, 22.72, 21.65, 21.32, 18.01 ppm; LR-ESI-MS *m/z* 299 [M + H]^+^; HR-EI-MS *m/z* 298.1569 [M]^+^ (calculated for C_19_H_22_O_3_ 298.1570).

To a stirred solution of **7b** (64 mg, 0.18 mmol) in NMP (2 mL), 1-dodecanethiol (0.13 mL, 0.54 mmol) and NaOH (43 mg, 1.08 mmol) were added. The reaction mixture was stirred for 1 h at 120 °C under microwave irradiation, and then quenched with 1N HCl (aq.) after cooling. The mixture was extracted using ethyl acetate and the combined organic phase was dried over MgSO_4_ and filtered. Volatiles were removed under reduced pressure, and the residue was purified by SiO_2_ chromatography to afford leotiomycene B (**1b**, 47 mg, 76%) as a white solid; Rf 0.19 (dichloromethane:methanol = 10:1); ^1^H NMR (500 MHz, Chloroform-d) δ = 11.48 (s, 1H), 6.55 (s, 1H), 6.43 (s, 1H), 6.36 (s, 1H), 6.20 (s, 1H), 5.34 (br s, 1H), 5.16 (t, *J* = 7.2 Hz, 1H), 3.24 (d, *J* = 7.2 Hz, 2H), 2.57 (s, 3H), 2.26 (s, 3H), 1.70 (s, 3H), 1.69 (s, 3H) ppm; ^13^C NMR (126 MHz, Chloroform-d) δ = 175.02, 166.29, 164.11, 155.99, 152.40, 145.21, 138.32, 135.14, 121.36, 117.07, 114.69, 114.05, 112.44, 105.14, 102.00, 25.89, 24.52, 23.09, 21.25, 17.95 ppm; LR-ESI-MS *m/z* 341 [M − H]^−^; HR-EI-MS *m/z* 342.1467 [M]^+^ (calculated for C_20_H_22_O_5_ 342.1468).

## 4. Conclusions

A microwave-assisted copper-catalyzed diaryletherification protocol was developed for the cross-coupling of aryl halides and sterically hindered *ortho*-prenylated phenols. The optimized method entailing microwave irradiation of a copper and bidentate ligand system (10 mol% each of CuI and N-(2-fluorophenyl)picolinamide with 2 equivalents of K_3_PO_4_ in CH_3_CN at 120 °C under microwave irradiation for 0.5 h) was successfully applied for the synthesis of bioactive prenylated diresorcinols, diorcinol I and leotiomycene B. Notable advantages of microwave-assisted catalysis include operational simplicity and rapid heating, as well as shorter reaction times and higher chemical yields. In addition, the highly regioselective prenylation of phenol was achieved for the preparation of *ortho*-prenyl phenol by directed lithiation and subsequent alkylation. This study provides importance for the development of various prenylated diresorcinols as chemical probes or drugs with novel modes of action. Furthermore, considering that prenylated benzenoids are biosynthetic precursors of various polycyclic natural products, this synthetic route can potentially be expanded to more complex bioactive compounds.

## Figures and Tables

**Table 1:** Optimization of Cu-catalyzed diaryletherification.

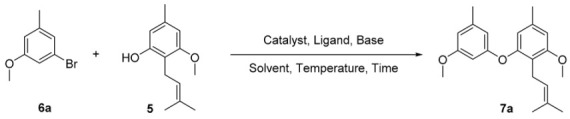							
Entry	Catalyst (mol%)	Ligand (mol%)	Base (eq)	Solvent	Temperature (°C)	Time (h)	Yield ^a^
1 [28]	CuO (250)	-	K_2_CO_3_ (1)	Pyridine	200 ^b^	1	ND ^c^
2 [29]	CuI (10)	Picolinic acid (20)	K_3_PO_4_ (2)	DMSO	90	30	ND
3	CuI (10)	Picolinic acid (20)	K_3_PO_4_ (2)	DMSO	120 ^b^	0.5	27%
4	CuI (10)	Picolinic acid (20)	K_3_PO_4_ (2)	DMSO	150 ^b^	0.5	50%
5	CuI (10)	Picolinic acid (20)	K_3_PO_4_ (2)	DMSO	200 ^b^	0.5	33%
6	CuI (10)	Pyrrole-2-carboxylic acid (20)	K_3_PO_4_ (2)	DMSO	150 ^b^	0.5	ND
7 [33]	Cul (10)	N-(2-fluorophenyl) picolinamide (10)	Cs_2_CO_3_ (2)	CH_3_CN	90	24	29%
8	Cul (10)	N-(2-fluorophenyl) picolinamide (10)	Cs_2_CO_3_ (2)	CH_3_CN	90 ^b^	0.5	40%
9	Cul (10)	N-(2-fluorophenyl) picolinamide (10)	Cs_2_CO_3_ (2)	CH_3_CN	120 ^b^	0.5	45%
10	Cul (10)	N-(2-fluorophenyl) picolinamide (10)	K_3_PO_4_ (2)	CH_3_CN	120 ^b^	0.5	88%
11	Cul (10)	N-(2-fluorophenyl) picolinamide (10)	K_3_PO_4_ (2)	CH_3_CN	150 ^b^	0.5	46%

^a^ Isolated yields. ^b^ The reaction was conducted under microwave irradiation. ^c^ Not detected.

## Data Availability

Not applicable.

## Supplementary Materials

The following supporting information can be downloaded at: https://www.mdpi.com/article/10.3390/molecules28010062/s1, Copies of ^1^H and ^13^C NMR spectra for **3**, **4**, **5**, **7a**, **1a** (Diorcinol I), **7b**, and **1b** (Leotiomycene B), Table S1: ^1^H and ^13^C NMR data of **1a** in comparison with the reported data of Diorcinol I; Table S2: ^1^H and ^13^C NMR data of **1b** in comparison with the reported data of Leotiomycene B.
