# Supplementary material for: Microwave-Assisted Cu-Catalyzed Diaryletherification for Facile Synthesis of Bioactive Prenylated Diresorcinols
Source: Molecules. 2022 Dec 21;28(1):62. doi: 10.3390/molecules28010062 (PMC9821922; doi:10.3390/molecules28010062)
Supplement: Supplementary file 1 [file molecules-28-00062-s001.zip › molecules-2102849-SI.pdf]

Supplementary Materials

Microwave-assisted Cu-catalysed diaryletherification for facile synthesis of bioactive prenylated diresorcinol

Seoyoung Jo[1,†], Bohun Kang, [1,2,†], and Jong-Wha Jung[1,2,*]

[1] College of Pharmacy, Research Institute of Pharmaceutical Sciences, Kyungpook National Univeristy, Daegu 41566, Republic of Korea
[2] Vessel-Organ Interaction Research Center, Kyungpook National Univeristy, Daegu 41566, Republic of Korea

[†] These authors equally contributed to this work.

* Correspondence to J.-W. Jung (Email: jungj@knu.ac.kr, Phone: +82-53-950-8578)

Contents

Copies of ^{1}H and ^{13}C NMR for **3**, **4**, **5**, **7a**, **1a** (Diorcinol I), **7b**, and **1b** (Leotiomycene B)

Table S1. ^{1}H and ^{13}C NMR data of **1a** in comparison with the reported data of Diorcinol I

Table S2. ^{1}H and ^{13}C NMR data of **1b** in comparison with the reported data of Leotiomycene B

Compound **3**

^{1}H NMR ($CDCl_3$)

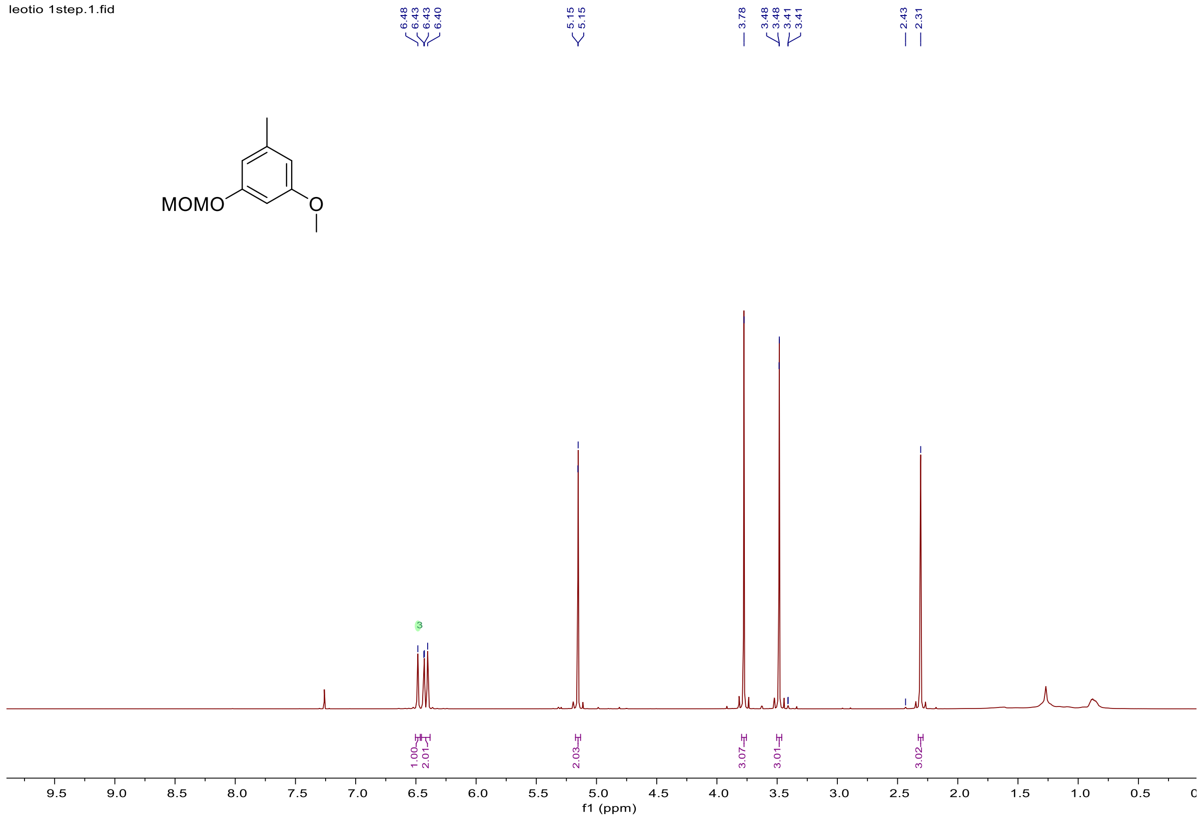

^{13}C NMR ($CDCl_3$)

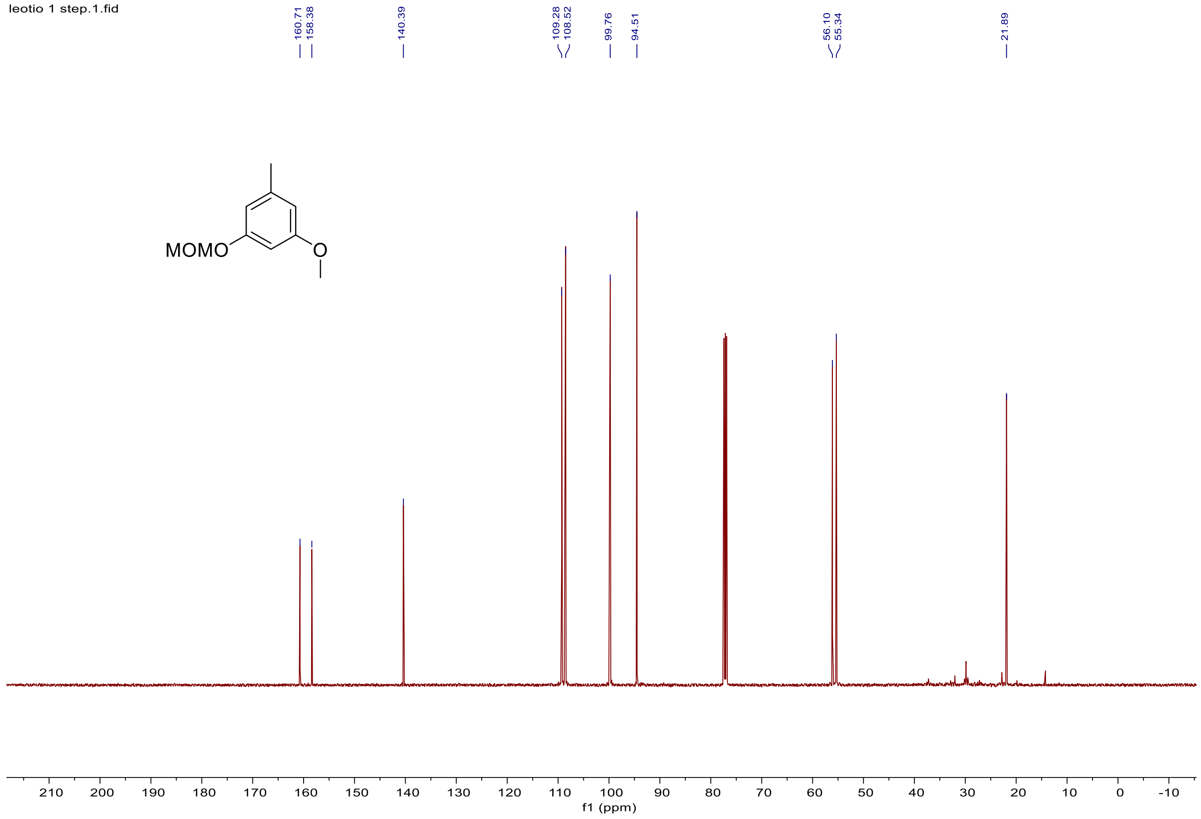

Compound **4**

^{1}H NMR ($CDCl_3$)

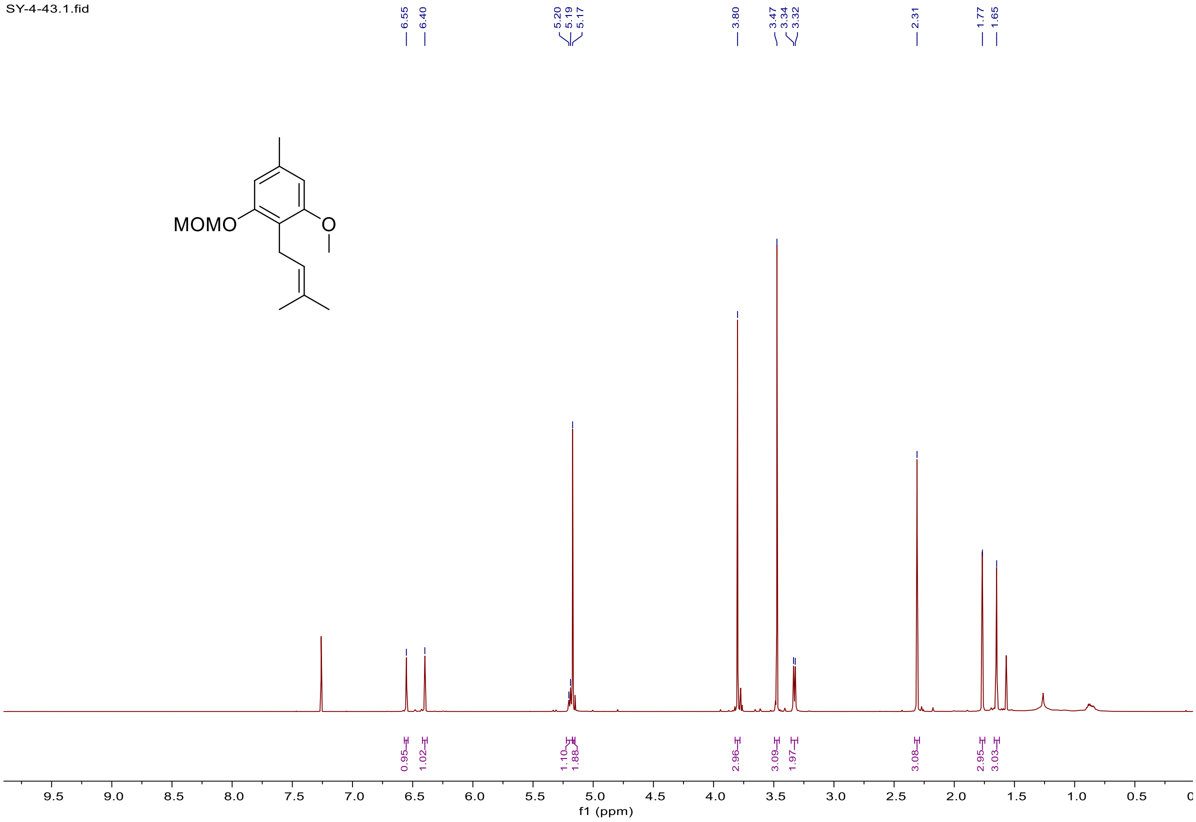

^{13}C NMR ($CDCl_3$)

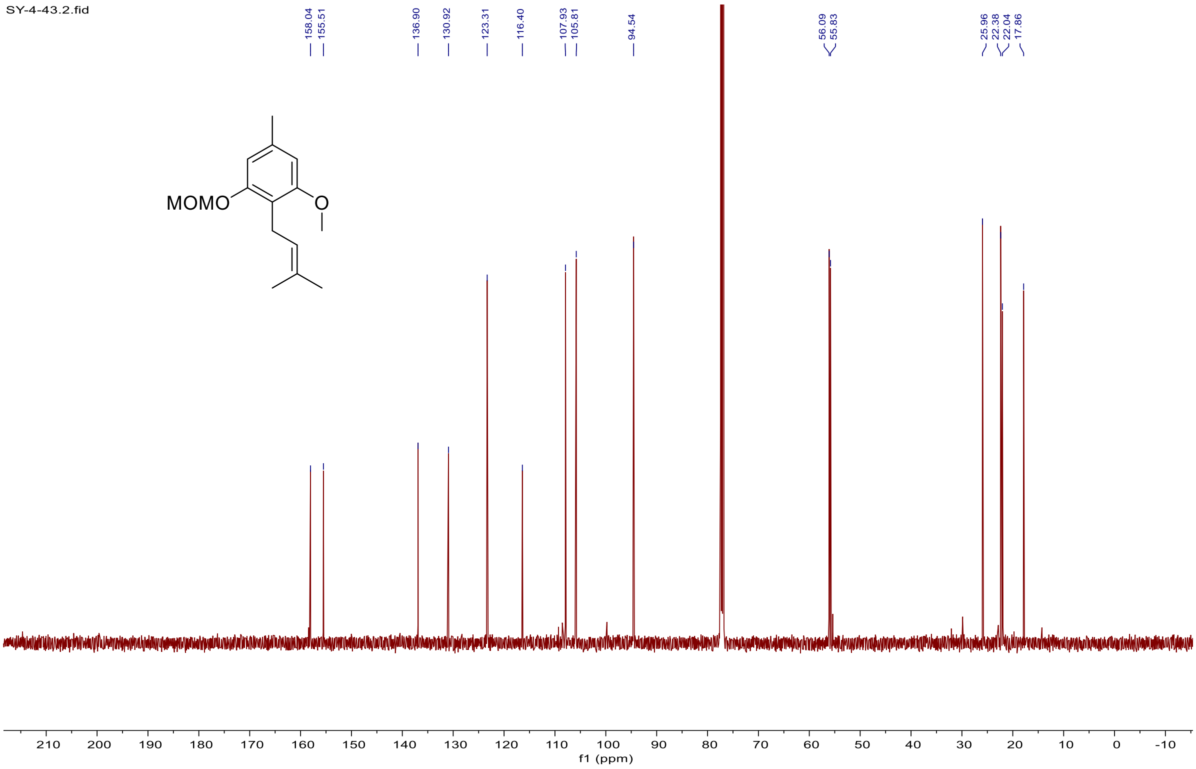

Compound **5**

^{1}H NMR ($CDCl_3$)

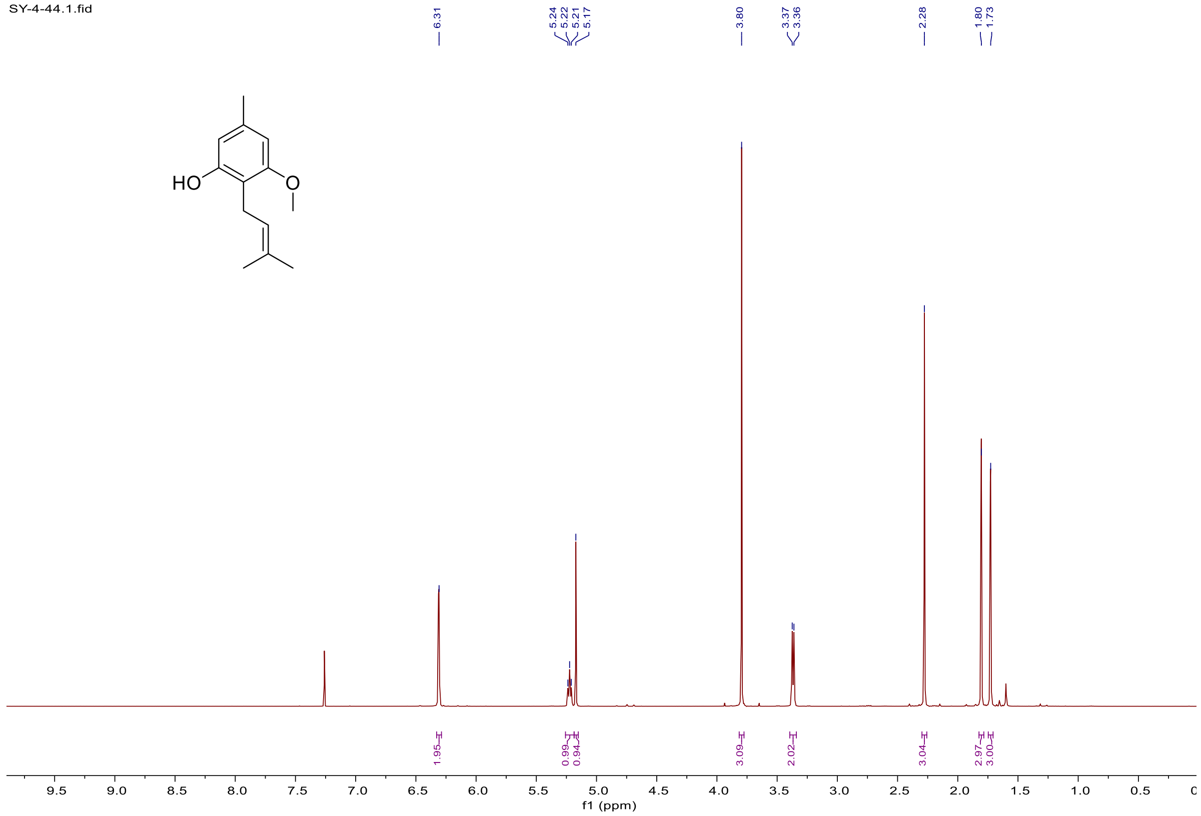

^{13}C NMR ($CDCl_3$)

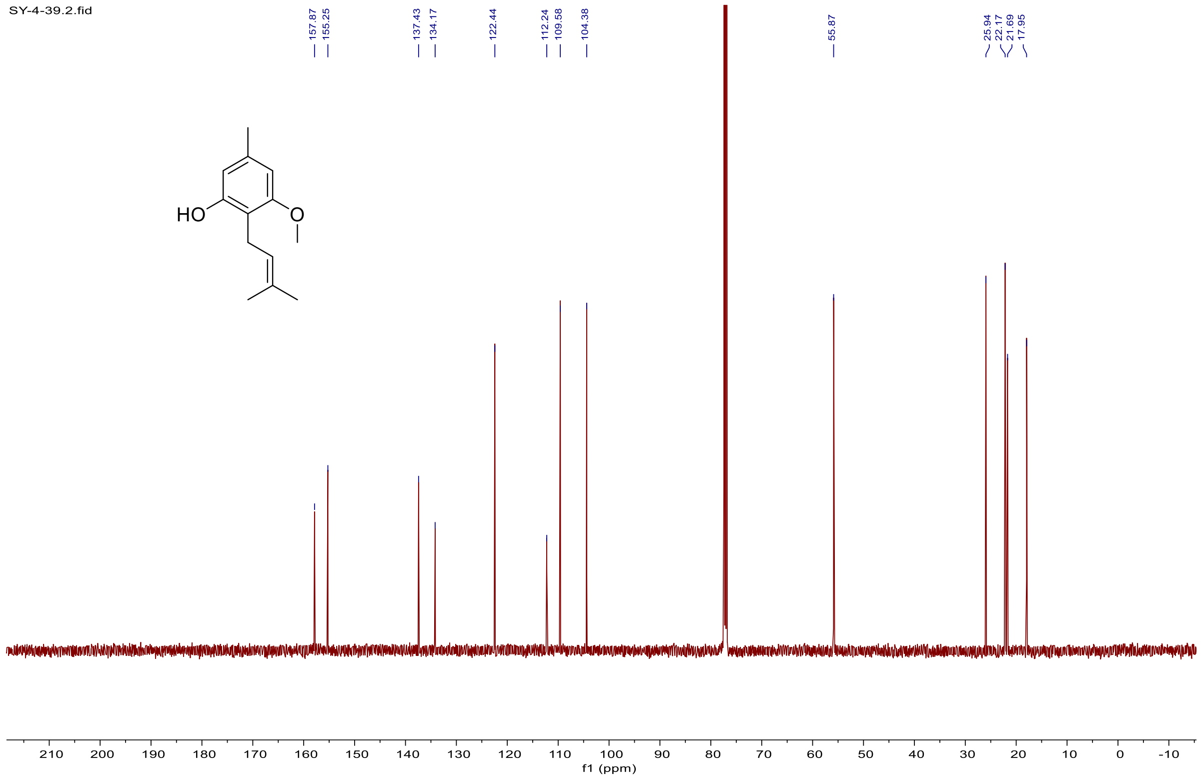

Compound **7a**

^{1}H NMR (CDCl$_3$)

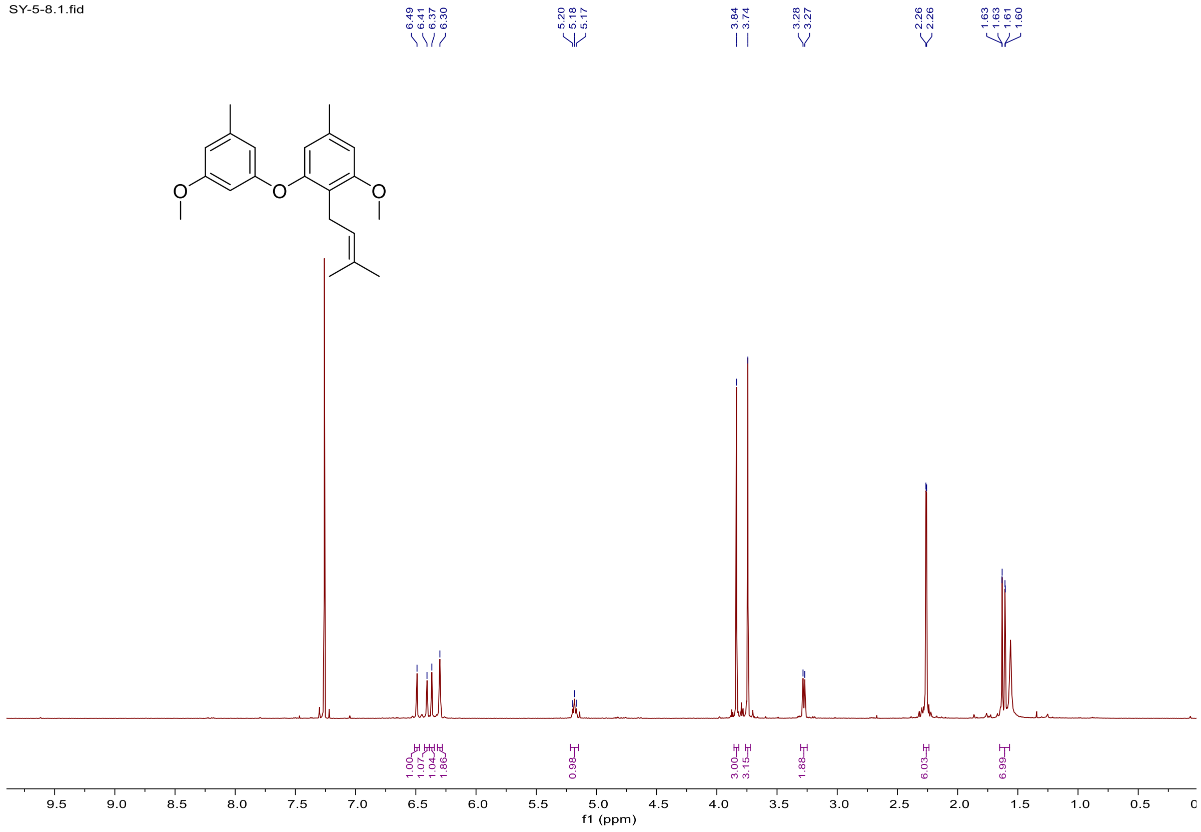

^{13}C NMR (CDCl$_3$)

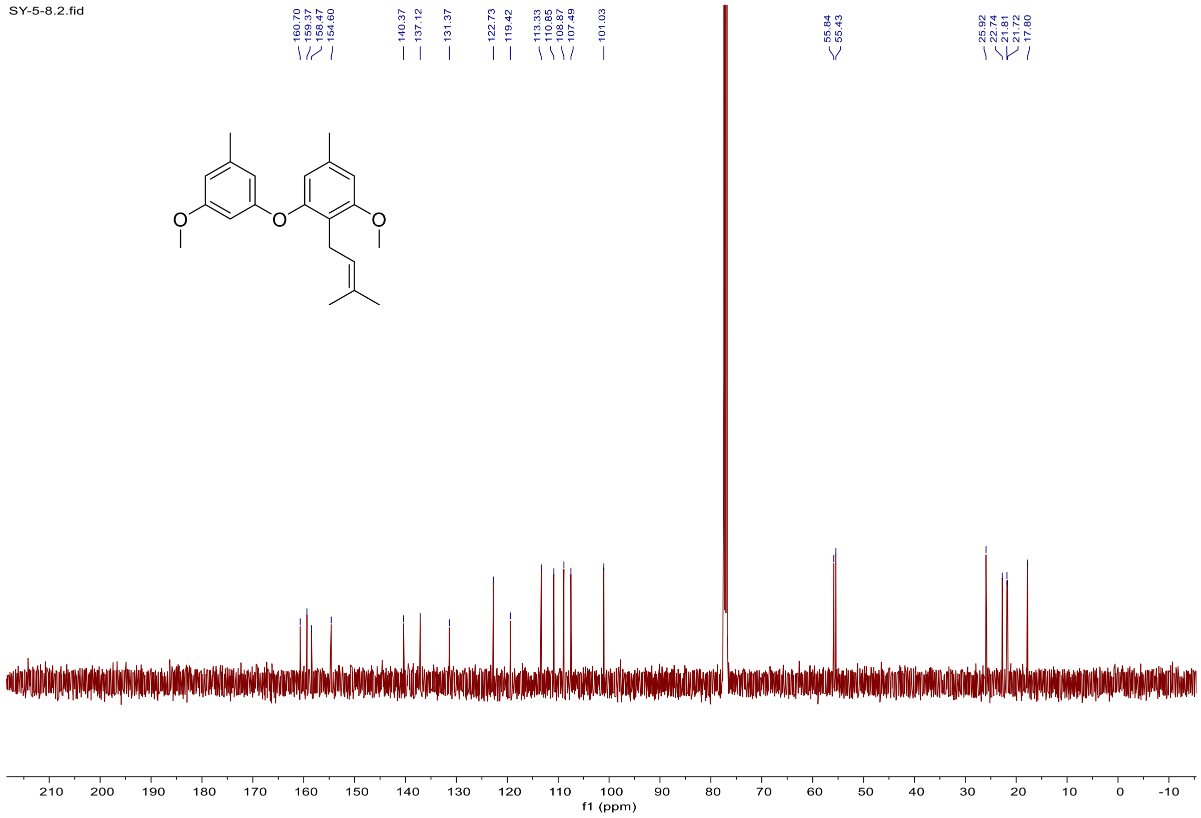

Compound **1a** (Diorcinol I)

^{1}H NMR (DMSO-d6)

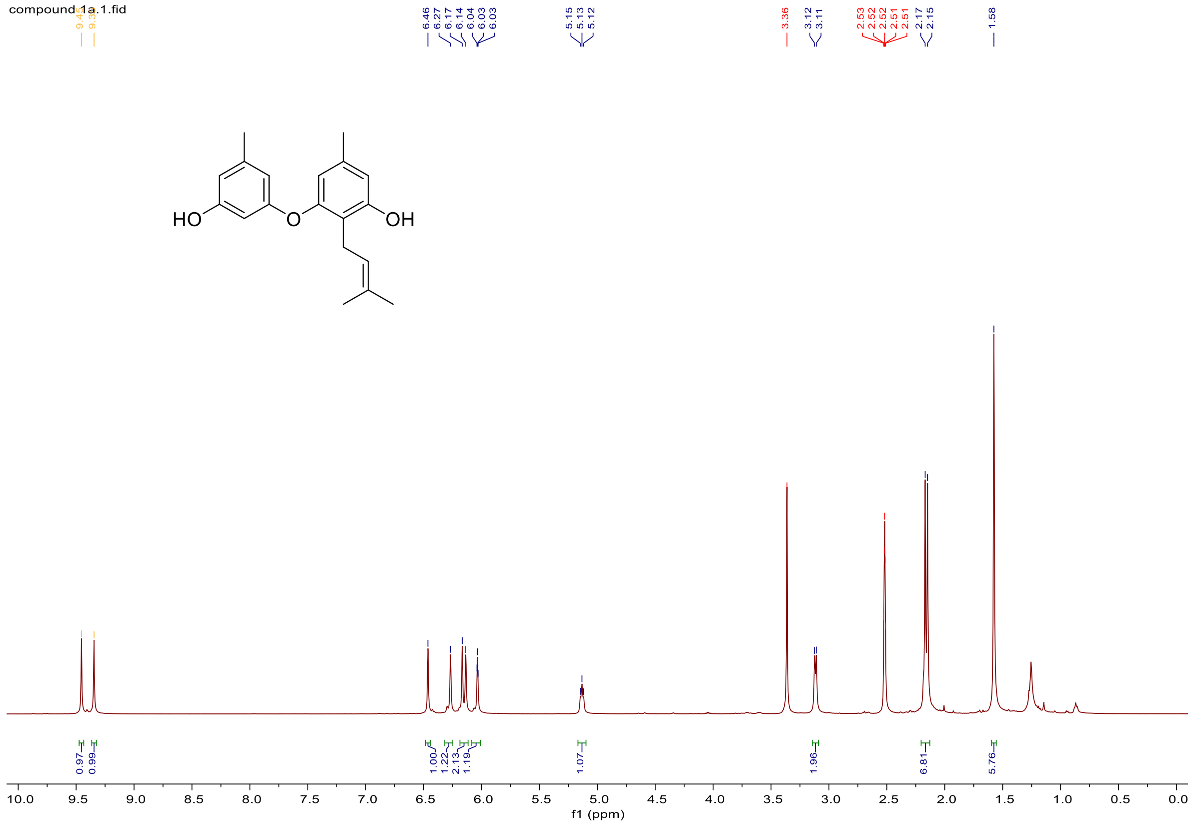

^{13}C NMR (DMSO-d6)

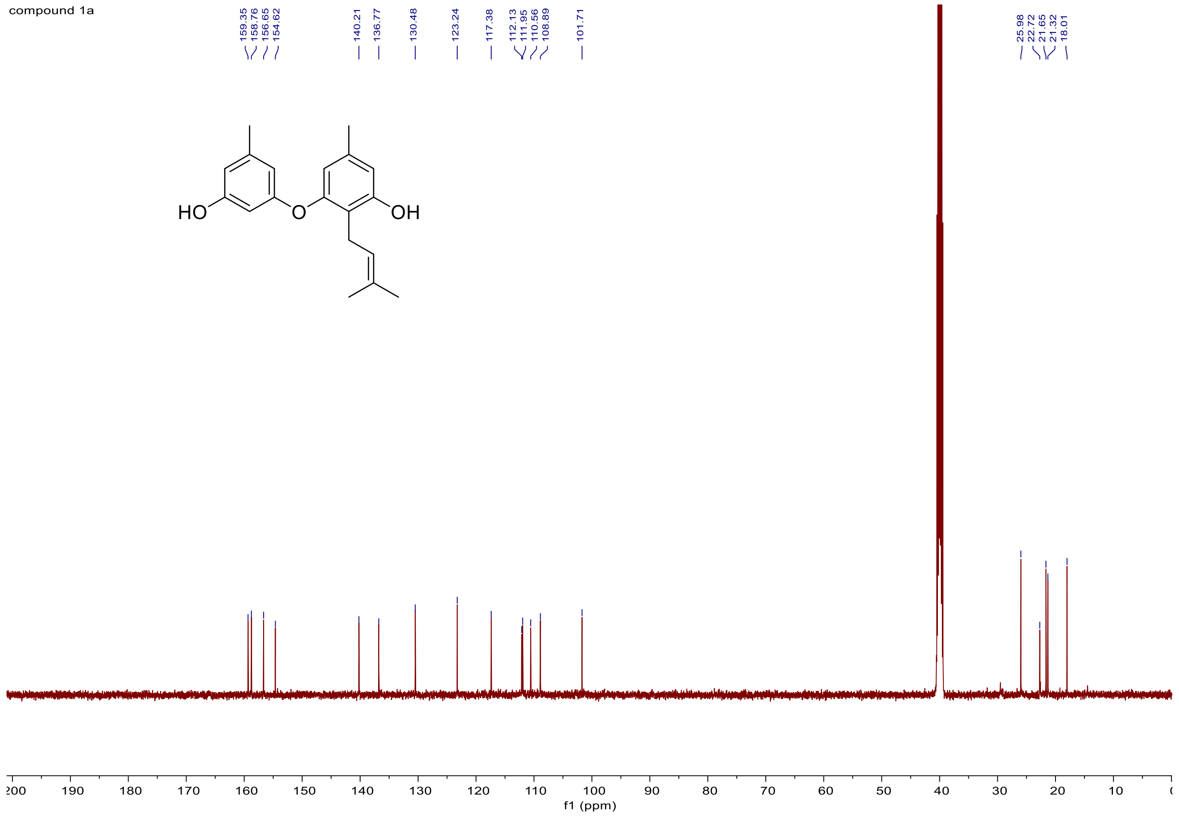

Compound **7b**

^{1}H NMR ($CDCl_3$)

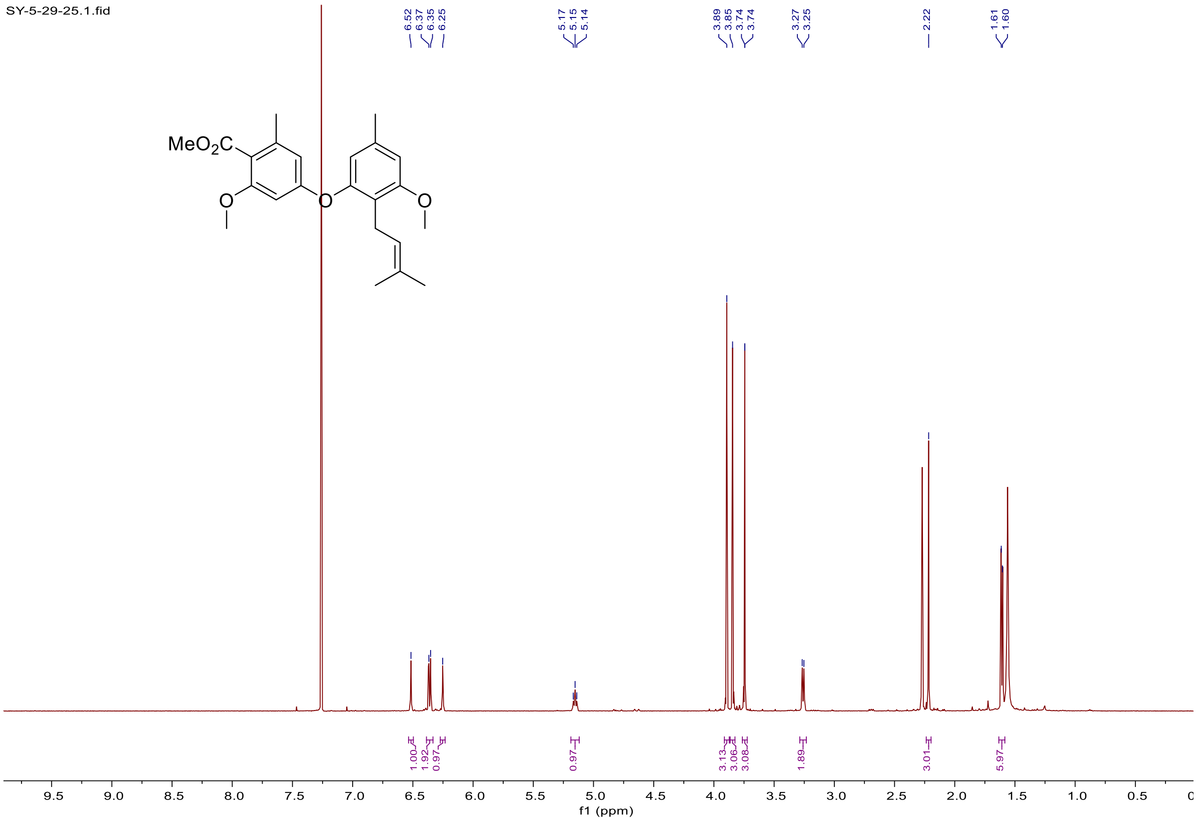

^{13}C NMR ($CDCl_3$)

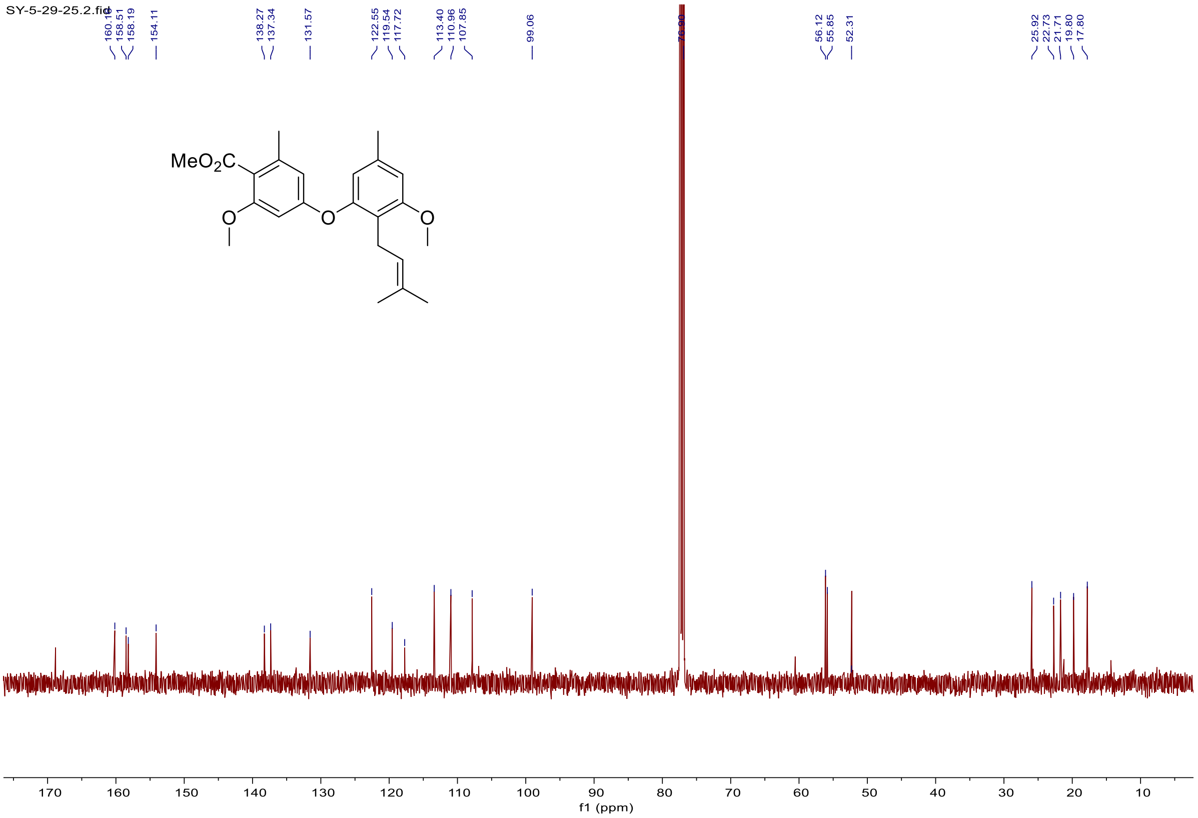

Compound **1b**

^{1}H NMR ($CDCl_3$)

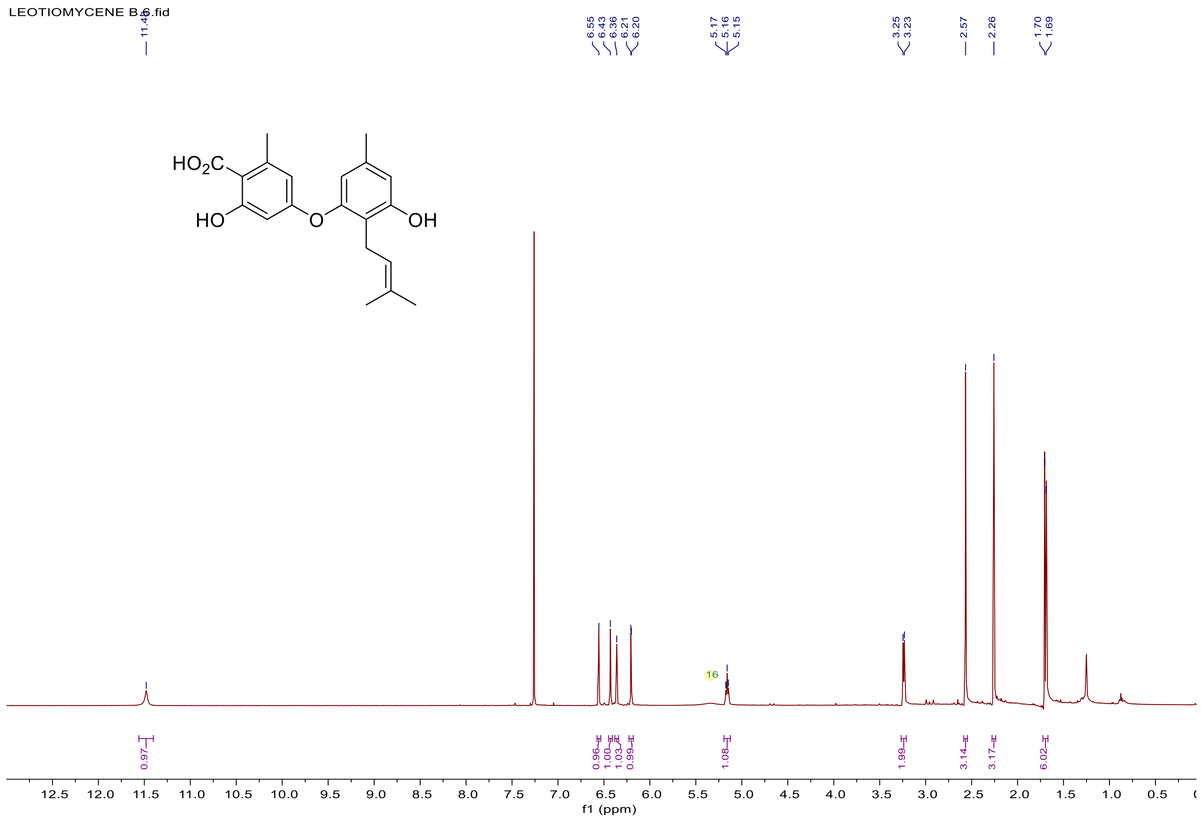

^{13}C NMR ($CDCl_3$)

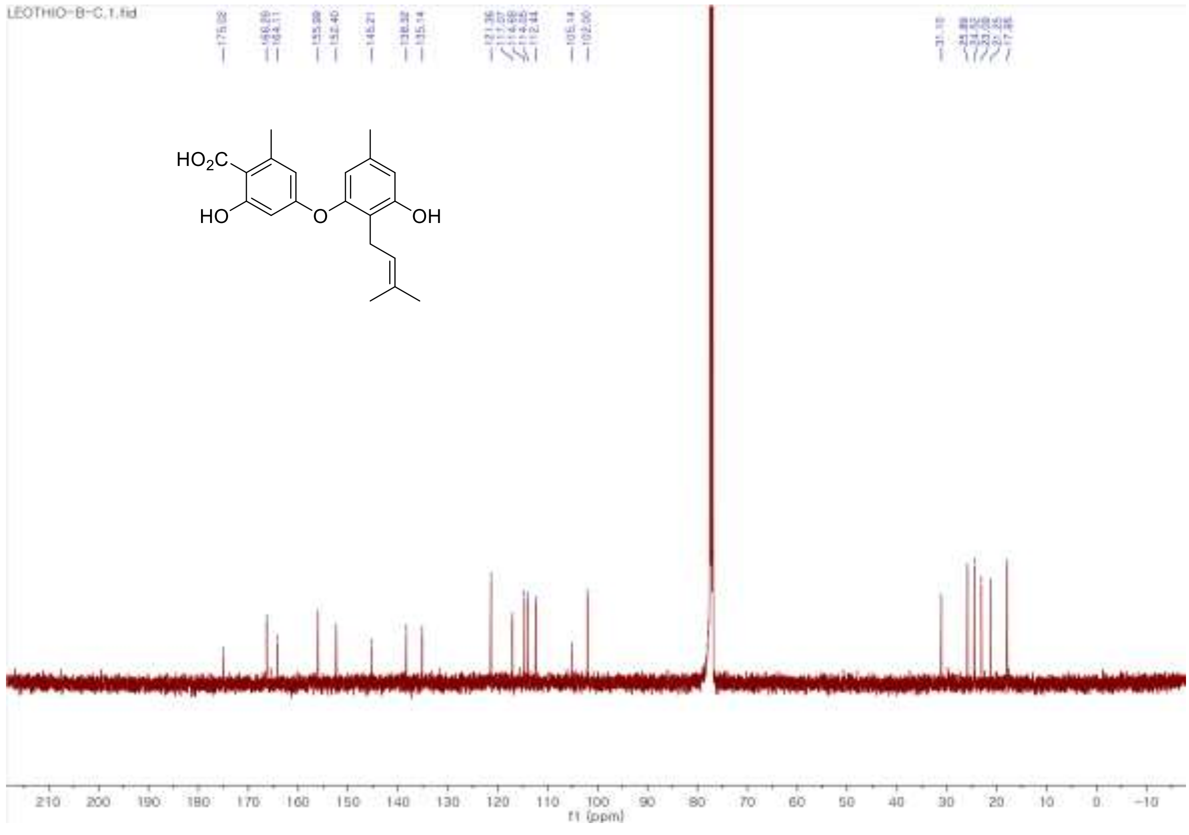

Table S1. ^{1}H and ^{13}C NMR data of **1a** in comparison with the reported data of Diorcinol I

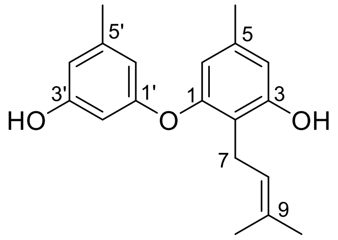

Position	**1a** (DMSO-d6)		Diorcinol I[a] (DMSO-d6)	
	δ (H)	δ (C)	δ (H)	δ (C)
1		154.62		154.2
2		117.38		117.0
3		156.65		156.3
4	6.46 (s)	112.13	6.45 (s)	111.7
5		136.77		136.3
6	6.17 (s)	111.95	6.15 (s)	111.5
1’		159.35		158.9
2’	6.03 (s)	101.71	6.02 (s)	101.3
3’		158.76		158.4
4’	6.27 (s)	110.56	6.25 (s)	110.2
5’		140.21		139.8
6’	6.14 (s)	108.89	6.12 (s)	108.5
5-Me	2.15 (s)	21.32	2.13 (s)	20.9
5’-Me	2.17 (s)	21.65	2.15 (s)	21.2
7	3.11 (d, J = 7.3 Hz)	22.72	3.10 (d, J = 7.2 Hz)	22.3
8	5.13 (t, J = 7.4 Hz)	123.24	5.12 (t, J = 7.2 Hz)	122.8
9		130.48		130.1
10	1.58 (s)	25.98	1.56 (s)	25.6
11	1.58 (s)	18.01	1.56 (s)	17.6
3-OH	9.45 (s)		NR[b]	
3’-OH	9.35 (s)		NR[b]	

[a]LI, Xiao-Bin, et al. Identification and biological evaluation of secondary metabolites from the endolichenic fungus Aspergillus versicolor. Chemistry & Biodiversity, 2015, 12.4: 575-592. [b]Not reported.

Table S2. ^{1}H and ^{13}C NMR data of **1b** in comparison with the reported data of Leotiomycene B

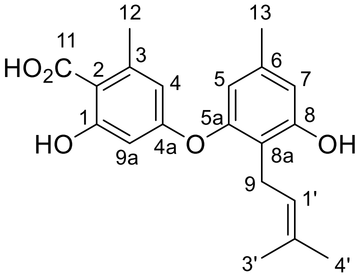

Position	**1a** ($CDCl_3$)		Leotiomycene B[b] ($CDCl_3$)	
	δ (H)	δ (C)	δ (H)	δ (C)
1		166.29		166.3
2		105.14		105.0
3		145.21		145.3
4	6.36 (s)	112.44	6.37 (d, J = 2.4 Hz)	112.5
4a		164.11		164.2
5	6.43 (s)	114.69	6.43 (br s)	114.7
5a		152.40		152.3
6		138.32		138.4
7	6.55 (s)	114.05	6.56 (br s)	114.1
8		155.99		156.0
8a		117.07		117.0
9	3.24 (d, J = 7.2 Hz)	23.09	3.24 (d, J = 7.1 Hz)	23.1
9a	6.20 (s)	102.00	6.20 (d, J = 2.4 Hz)	102.0
11		175.02		175.0
12	2.57 (s)	24.52	2.57 (s)	24.6
13	2.26 (s)	21.25	2.26 (s)	21.3
1’	5.16 (t, J = 7.2 Hz)	121.36	5.16 (tm, J = 7.1 Hz)	121.3
2’		135.14		135.3
3’	1.70 (s)	17.95	1.71 (br s)	18.0
4’	1.69 (s)	25.89	1.69 (br s)	25.9
1-OH	11.48 (s)		11.44 (s)	
8-OH	5.34 (br s)		5.26 (br s)	

[b]PAGUIGAN, Noemi D., et al. Prenylated diresorcinols inhibit bacterial quorum sensing. Journal of Natural Products, 2019, 82.3: 550-558.
